# SIMS: A Hybrid Method for Rapid Conformational Analysis

**DOI:** 10.1371/journal.pone.0068826

**Published:** 2013-07-23

**Authors:** Bryant Gipson, Mark Moll, Lydia E. Kavraki

**Affiliations:** 1 Department of Computer Science, Rice University, Houston, Texas, United States of America; Bioinformatics Institute, Singapore

## Abstract

Proteins are at the root of many biological functions, often performing complex tasks as the result of large changes in their structure. Describing the exact details of these conformational changes, however, remains a central challenge for computational biology due the enormous computational requirements of the problem. This has engendered the development of a rich variety of useful methods designed to answer specific questions at different levels of spatial, temporal, and energetic resolution. These methods fall largely into two classes: physically accurate, but computationally demanding methods and fast, approximate methods. We introduce here a new hybrid modeling tool, the Structured Intuitive Move Selector (sims), designed to bridge the divide between these two classes, while allowing the benefits of both to be seamlessly integrated into a single framework. This is achieved by applying a modern motion planning algorithm, borrowed from the field of robotics, in tandem with a well-established protein modeling library. sims can combine precise energy calculations with approximate or specialized conformational sampling routines to produce rapid, yet accurate, analysis of the large-scale conformational variability of protein systems. Several key advancements are shown, including the abstract use of generically defined *moves* (conformational sampling methods) and an expansive probabilistic conformational exploration. We present three example problems that sims is applied to and demonstrate a rapid solution for each. These include the automatic determination of “active” residues for the hinge-based system Cyanovirin-N, exploring conformational changes involving long-range coordinated motion between non-sequential residues in Ribose-Binding Protein, and the rapid discovery of a transient conformational state of Maltose-Binding Protein, previously only determined by Molecular Dynamics. For all cases we provide energetic validations using well-established energy fields, demonstrating this framework as a fast and accurate tool for the analysis of a wide range of protein flexibility problems.

## Introduction

Proteins lie at the root of nearly all biological processes and often accomplish functions through conformational changes in their structure. An understanding of conformational variability therefore would provide valuable insight into protein function, in addition to aiding pharmaceutical drug design – given that drug binding sites often become exposed as the result of conformational changes. The development of computational methods for the analysis of protein flexibility has a long history [1]–[3], with several broad classes of analytical frameworks having been developed over the years. Rigorously accurate, yet computationally demanding physics-based methods were among the first and best attempts to address such questions by solving equations of motion defined by a particular protein system. While definitive for high-resolution and physically accurate interpretations, such methods have typically been limited by protein size due to computational complexity [4], [5]. More recently, a class of methods has been developed that use approximations to quickly provide analytical insight into key biological processes. This class includes a broad range of methods, such as coarse-grained energy calculations [6], multi-scale models [7] and alternative representations of flexibility, such as Normal Mode Analysis [8]–[11] and Dynamic Elastic Networks [12]–[14], among others.

Recently, a hybrid class of mechanistic approaches has gained traction for the analysis of molecular structures, inspired by the field of robotic motion planning [15], [16]. Such methods attempt to bridge the divide between the above classes and are capable of using highly accurate energetics for representation, while additionally employing long-range moves for conformational exploration. In the motion planning inspired approach, molecules can be regarded as long articulated chains with atoms as links and bonds as joints. Using this representation, the energy of a particular protein conformation (computed using any available method) is used as a selection criterion during conformational exploration. Exploration can occur by sampling new conformations through the perturbation of known “good” conformations using any available move. The resulting conformation is checked for feasibility by the provided energy function. If it *is* feasible, it is added to the set of “good” conformations.

The central strength in motion planning-inspired approaches lies in their ability to adaptively guide exploration based on estimates of the density of known conformational samples. Typically, they use some notion of coverage to “push” the exploration away from well-explored conformations (i.e., redundant and highly similar sampled states) and towards unexplored parts of conformational space. This process can rapidly lead to an increasingly accurate approximation of the local conformational flexibility of a protein and typically operates orders of magnitude faster than a random thermodynamic walk [17].

While motion planning-inspired methods are not designed to specifically model physically accurate molecular motions, they are capable of rapidly producing a *representative* approximation of the local conformational variability of a protein under study. Motion planning has recently been applied to a wide range of biologically important subjects including rna folding [18], protein loop modeling [19]–[22], protein folding/binding [23]–[25], conformational flexibility [17], [26] and conformational transitions [27], [28], among others.

This paper introduces a highly general framework, the Structured Intuitive Move Selector (sims), used for the automatic or expert-guided discovery and analysis of the conformational variation of arbitrary protein molecules. We demonstrate several key advances that allow us to revisit and significantly improve upon results obtained by earlier robotics based methods. We show that sims can identify and use “active” residues (i.e., residues most likely to be involved in conformational transitions) in the exploration of hinge-based systems such as Cyanovirin-N. sims is also shown to be capable of identifying significant, long-range, correlated changes in Ribose Binding Protein and is shown to discover a “hidden” (experimentally unobserved) conformation of Maltose-Binding Protein, at a fraction of the computational cost of Molecular Dynamics (md) simulations.

The contributions of sims as a method can be summarized as follows. It adopts a state-of-the-art motion planning algorithm for conformational sampling. It also introduces *structured local move selection*: a unified approach to intelligently perturbing conformations to obtain new conformations that combines loop sampling, energy minimization, and dihedral angle sampling. Thanks to the level of abstraction the approach provides, other moves can easily be added. The moves are applied to protein “fragments,” groups of possibly non-contiguous residues meant to approximate functional, structural or dynamically correlated regions of the protein. The decomposition of a protein into fragments can be done automatically, but allows an expert user to define fragments as well. Finally, sims is designed to run in parallel and requires only minimal communication, allowing it to be run on a large scale.

### Generic planning algorithms for conformational sampling

The initial ideas regarding the application of robotic motion planning to proteins were introduced in [29] and used the Probabilistic Roadmap Method (prm) [30] to build a roadmap for the motion of a small ligand around a protein. The roadmap is a graph representation of conformational transitions, where each node represents a conformation and each edge a transition between two conformations. During the construction of such roadmap, an energy function is used to verify whether a conformation or transition is biophysically plausible. If a conformation or transition is not feasible, it is simply discarded. These initial results followed from significant advances in motion planning around the same time. Rather than developing algorithms for exact, optimal solutions (which is, computationally, prohibitively expensive), motion planning research shifted in the 1990s to the development of sampling-based planning algorithms, which have been very successful in practice and are currently the main way to plan paths for complex robots. Subsequent work on applying sampling-based motion planning to conformational sampling [31] introduced the stochastic roadmap simulation, established the connection with Monte Carlo methods and dealt with problems involving conformations of much larger protein molecules. prms for the computation of folding pathways given the 3D structure of the protein have also been investigated at length in a series of papers that span a decade (see [32] for a detailed discussion). This line of work has provided important insights into the order of formation of secondary structures that agree with experiments [24]. Two recent surveys [33], [34] provide an extensive overview of geometric and kinematic modeling of protein structures as well as the application of motion planning techniques for modeling protein motion. Below, we give a brief overview of such algorithms. The algorithm used in this paper will be described in more detail in *Methods*.

In recent years, specific motion planning algorithms have seen significantly increased use with regard to the protein flexibility problem. In particular, the application of the Rapidly-exploring Random Tree (rrt) algorithm [35] to molecular simulations has expanded dramatically. This algorithm attempts to explore protein conformational variability by growing a tree of conformations, starting from a known structure. The algorithm iteratively samples a uniformly random conformation, finds the most similar conformation in the tree, and extends the tree from this conformation towards the random conformation. The transitions between conformations are typically obtained by simple interpolation of the Degrees of Freedom (dofs). Protein loops have been successfully analyzed using this method [19] (though generating the “random” loop conformations required special attention). More recently, long-range protein conformational analysis has been performed [27]. To reduce the computational cost, the authors used a priori information in the form of “predicates” to solve certain highly constrained planning problems (see *Results*). This work highlighted that rrt-based approaches are difficult to scale up to proteins with hundreds and hundreds of thousands of dofs. Perhaps this is to be expected as protein conformations with uniformly random backbone angles almost always represent an unfolded protein, often with many steric clashes. Moving toward random conformations may therefore not represent an ideal method for efficiently exploring conformational changes. In our implementation we use a recently proposed alternative to rrt called Kinodynamic Planning by Interior-Exterior Cell Exploration (kpiece) [36] which is a member of a class of expansive planners [37]. This specific algorithm will be described in more detail in *Methods*. Like rrt, expansive planners grow a tree of conformations. *Unlike* rrt, these planners use estimates of local state density to push tree growth towards unexplored regions of the conformational space (i.e., regions with low density). While rrt and expansive planners may seem somewhat similar, they exhibit markedly different behavior in practice, especially as the number of dofs increases.

The mechanism that expansive planners use to create a new conformation in a neighborhood of a previously generated conformation can incorporate techniques that increase the probability of sampling energetically feasible conformations. In this work, we define a library of moves that each individually has been used in prior work for conformational sampling, but not in an integrated way as is done here. This library includes: energy minimization, loop sampling [22], random dihedral angle perturbation, and “natural moves” similar to [38]. An expansive planning algorithm thus grows a tree of conformations that preferentially expands away from a set of starting states towards less-explored regions of the energetic landscape.

### Proteins and energy functions

Typically, important biological functions are performed by folded, compact proteins existing in one of a few stable conformations available at cellular conditions. Stable conformational ensembles represent groups of protein states at low free energy and are typically associated with basins about the minima of the potential energy field [39], [40]. An understanding of protein stability therefore requires an accurate notion of potential energy. Many potential energy functions have been proposed (see [41], [42] for detailed discussions), typically for md simulations. Energy calculation typically represents the largest computational cost when modeling changes in proteins, and the use of the above models can prove prohibitively expensive. While sims is not restricted to any particular energy function, in this paper we rely on the Rosetta [43] library, which contains efficient implementations of many full-atom energy models, striking a good balance between accuracy and speed of computation.

As in earlier work, “active” dofs are limited to the 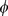 and 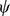 backbone angles [17], [27], [28], [44] and side-chain positions are automatically determined by Rosetta's side-chain minimization protocol [27]. We used the Rosetta “score12_full” energy function for the experiments, which provides an atomic representation of all atoms, implicitly modeling solvation and related energetic terms. At the end of the paper we show energy validations against the Amber99 [45] force field as implemented by the software package mmtk [46], showing excellent agreement for all results and demonstrating that Rosetta energy calculations were sufficiently accurate for the studies in this paper.

### Motivating problems

To demonstrate the range and generality of analysis that sims can provide, we present three important problems often encountered in computational biology. Below, we introduce protein systems that are shown to characterize these problems and in later sections present results for each. The first two problems have been previously studied by a related robotic motion planning-inspired method [27], and were specifically chosen to enable a direct comparison. These problems involve the use and determination of *active* dofs, especially in the context of previously defined (and possibly incomplete or inaccurate) expert knowledge. By *active* dofs we mean a set of dihedral angles from a range of residues that represent the minimal set of angles that must change in order to allow a particular type of conformational transition to occur. The final problem has been investigated primarily by md and, though sims is not designed as an alternative to such methods, is presented as a case where sims can be used to replicate valuable conformational insights quickly and automatically.

*Cyanovirin-N* (cvn) is a two-domain bacterial anti-viral protein, capable of binding to the surface sugars of a range of viruses including hiv. cvn is known to occur in monomeric [47] and domain-swapped [48] forms, with the domain-swapped conformation found to posses higher anti-viral affinity than the monomer [49]. It is known that these two conformations co-exist in solution [49] and transitions between them were previously computed [27], but depended on expert knowledge.

*Ribose-Binding Protein* (rbp) is part of a ribose transport system in bacteria and is additionally involved in chemotaxis. It is composed of two domains connected by a hinge formed by three well-separated loops. Both closed [50] and open [51] forms of rbp are known for this system. While the active dofs in this system are known to occur almost exclusively in the hinge region, domain movement can only occur as a result of coordinated motion among the three loop regions. The two forms of rbp are separated by just over 4Å, and the required domain transition seems deceptively simple; a visually convincing transition between the forms can be quickly computed with, e.g., ucsf Chimera [52]. However, solving this problem in an energetically feasible manner that preserves the kinematic bond structure of the protein is quite challenging. As a result, prior work [27] relied on artificial distance restraints to maintain “reasonable” structures during sampling.

*Maltose-Binding Protein* (mbp) is a well-studied bacterial protein involved in chemotaxis, biosensing, the maltose/maltodextrin system of E. coli and is also often used as an affinity tag in protein purification and expression. mbp is important for biological and experimental reasons. Though many structures have been determined for mbp by X-Ray crystallography and other methods, most of these fall into the classes of “open” and “closed” states, as determined by the degree of bending between the C and N terminal domains. A third “hidden” semi-closed intermediate was recently determined by accelerated md [53], though it had been previously indicated by nmr [54] and earlier computational studies [55]. While ligand binding is known to drive conformational change, nmr [54] studies have shown that mbp exists in solution in a mixture of these states. An analysis of the available set of mbp proteins [56]–[84] shows a high degree of spatial and torsional variation for essentially all residues, excepting several short stretches in core helical regions. Further, the difference between open and closed forms is one of tightly constrained long-range “bending” occurring across the entire molecule, as opposed to simple rigid body changes in sub-domains, producing extensive side-chain interactions. This system represents a difficult challenge in that no clear set of active dofs exists and the motion is extremely coordinated.

## Methods

The central problem we address here is of how to vary the dofs of a protein in such a way that the energy never exceeds biologically feasible bounds when attempting to find low-energy conformational transformations between known states. As was done in prior work [17], [27], [28], [44], [85], we represent a conformation of a protein by just the backbone angles. The positions of side-chain atoms for any given conformation are determined by side-chain optimization and bond angles and lengths are always idealized. This representation significantly reduces the computational difficulty of the problem.

Below we first describe the primitive “moves” that will be used to perturb conformations. These moves typically do not affect the entire structure, but instead correspond to local changes. We propose a way to automatically define a collection of residue subsets called a *schema* on which the moves operate. Finally, the high-level planner maintains state density estimates which it uses to apply moves to conformations in relatively sparsely sampled parts of the conformational space.

### Structured move selection

Computationally generating new conformations based on known states involves applying some type of perturbation of the dofs of the system. We call such a perturbation a *move*. Many different types of moves have been proposed, including dihedral perturbations [86] and Normal Modes [8]–[11], as well as moves based on Dynamic Elastic Networks [12]–[14], [87], to name a few. In our method, moves can be applied to both small protein fragments (such as loop regions) and the whole structure. We use a *schema* to define subsets of dofs on which moves operate. Such a schema can automatically be constructed based on the structure of a protein. For example, one can define a subset for each domain, each secondary structure element or even each residue. Each residue can be part of multiple subsets. Often, an expert may wish to define additional subsets. For example, the three loop regions in rbp that connect two domains can form an additional subset, since motions of the dofs within that subset are highly coordinated. Note that a subset of residues does not need to correspond to a contiguous sequence of residues. Associated with each subset is a probability for selecting that subset for a move. These probabilities can be defined heuristically based on what is known from the literature about the relative flexibility of, e.g., secondary structure elements: flexible loop fragments will be sampled with a higher probability than more rigid alpha helices.

Associated with each subset is a probability distribution over the “allowed” moves. In our experiments described below we used the following moves:

#### Dihedral angle sampling

This is simply a uniformly random perturbation (up to 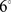) of each dihedral angle within a subset.

#### Loop sampling

Here, a random conformation of a loop region (or collection of loop regions) is generated, subject to the constraint that the endpoints of each loop are kept in the same position.

#### Rigid body movements

This type of move corresponds to a small displacement of one loop endpoint relative to another while maintaining the kinematic constraints of the loop. This move enables fast sampling of whole domain rearrangements.

#### Energy minimization

This move is applied with low probability to the entire protein since it is computationally expensive.

**Figure 1:**
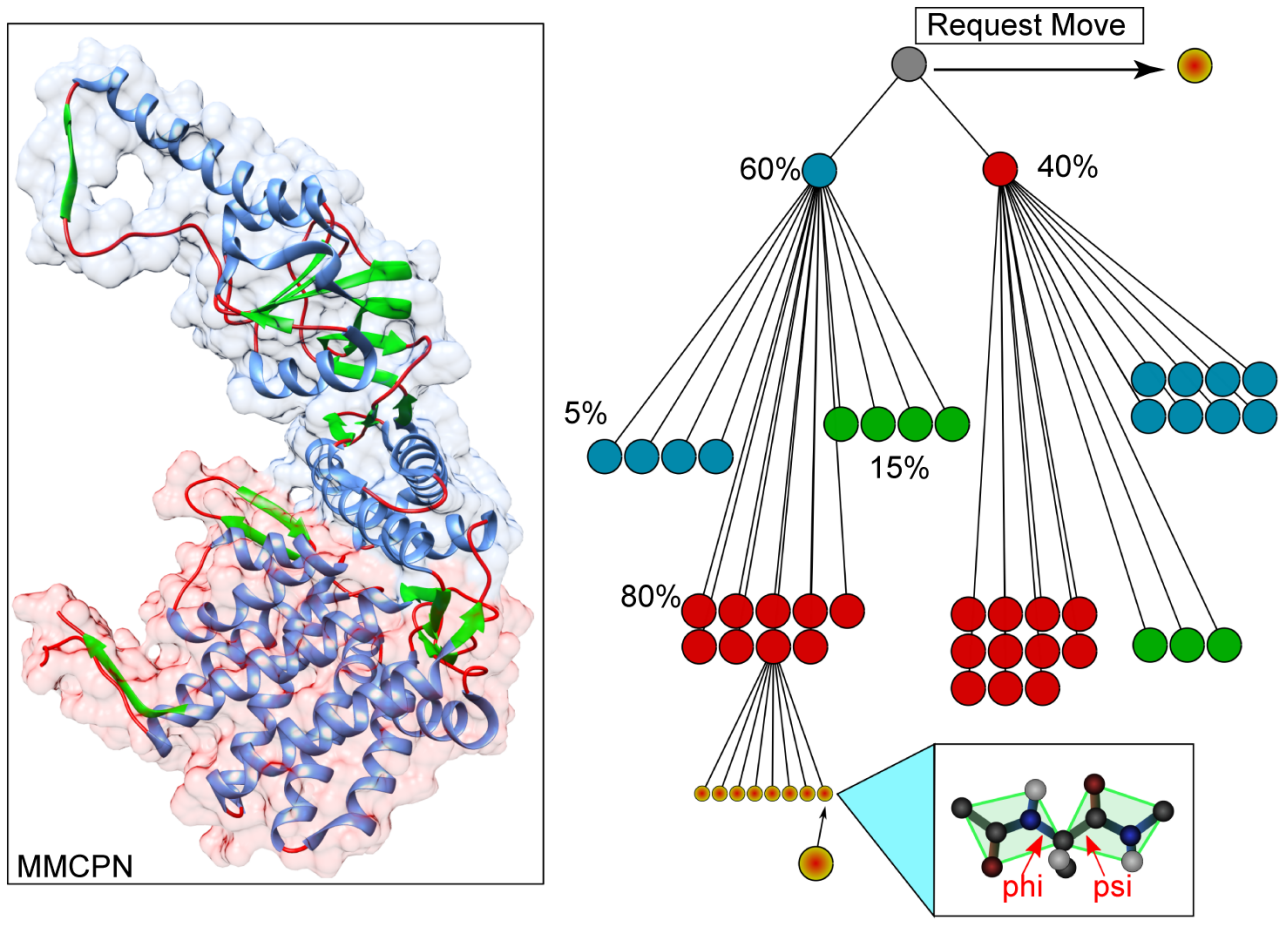
Example of a structured schema for an arbitrary molecule. Subsets of dofs are defined, along with the associated weighting (shown here as percentages) defining the relative probability of selection. Though this example is non-overlapping and hierarchical, any combination of possibly non-contiguous subsets are allowed in our implementation. In this example, a move is generically requested, and subsequently sampled probabilistically from the set containing all loop regions in the top (blue) region of the structure. The yellow circles represent possible moves.

A schematic overview of a schema and move selection is shown in Figure 1. Although the figure shows a hierarchical decomposition, this does not have to be the case (unlike [88]). As mentioned, a default schema can be automatically computed from the primary structure, but expert knowledge can easily be incorporated as well. Not only can extra subsets be defined, also the types of moves and the probabilities of selecting a move can be changed, if there exists prior knowledge about a suspected mechanism underlying some conformational change.

### Rosetta

The Rosetta Library [43] has been applied to a considerable number of protein systems and problems in recent years [89]–[93], due to its powerful algorithmic flexibility and extensive library of protocols for protein modeling. While not strictly dependent on the library, sims is able to take advantage of Rosetta for structure representation and modification as well as minimization and energy analysis. This allows any experiment performed with sims to be run in centroid mode or with the full atom representation mode, along with user-specified weightings to energy terms as needed (though the “score12_full” scoring function was used for all simulations presented here). Moreover, conformational sampling can be performed by taking advantage of the extensive library of moves available in Rosetta's sampling protocols, including minimization, ccd loop closure [94], and loop-sampling [95]. The sims moves described above have been implemented using Rosetta's moves. It is important to note, however, that any alternative representation or energy calculation library could have been used in its place.

### Efficient conformational sampling using a motion planning algorithm

The moves described above can be used by an expansive motion planning algorithm to grow a tree of conformations, where each conformation is derived from its parent through a move. Many robot motion planning algorithms have been proposed over the years, and many of them are implemented in a very abstract way in the Open Motion Planning Library (ompl) [96]. This level of abstraction makes it possible to adapt them for conformational exploration. While in robotics, a collision checker is often used to decide whether a robot configuration is valid, here we use an energy threshold as a criterion for accepting sampled conformations. We used ompl's default high-dimensional planner, called kpiece [36], for all experiments presented in this paper. kpiece has previously been shown to be very effective in high-dimensional spaces, including kinematic chains of rigid bodies – systems similar to proteins. We will give a brief description of kpiece algorithm below; for details see [36].

kpiece approximates the density of sampling of the conformational space through a projection of all the dofs. High-dimensional systems are often constrained to move on a low-dimensional manifold embedded in a high-dimensional space. Proteins are no exception: once proteins are folded the dofs are often very constrained. Using a low-dimensional projection allows for efficient estimation of sampling density. The default projection we have defined is a random, linear 2D projection of the cosines and sines of the dihedral angles. This projection is computed as follows. For a conformation with 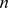 dihedral angles, a vector of size 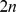 is computed with the cosines and sines of all angles. This vector is projected to a 2D point with a matrix 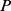 of size 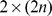. The matrix 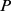 is constructed by first drawing its entries from a normal distribution with mean 0 and variance 1. Next, the first row is normalized to be of length 1. Finally, the second row is made orthogonal to row 1 and then also normalized. This process can be generalized to any 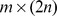 projection matrix. The projection is chosen randomly because (a) there is no natural choice of projection in general and (b) prior work has shown that a random projection often captures sample density quite well compared to an optimal or expert-chosen projection [97]. Given a 2D projection, all conformations can (for the purpose of density estimates) be represented by 2D points. kpiece defines a 2D grid and maintains a count of the number of conformations per grid cell. It then (1) samples a grid cell with probability inversely proportional to its density, (2) samples a conformation uniformly at random from that cell, (3) applies a random move selected in the manner described in the previous section, and (4) checks if the conformation's energy is below a user-specified threshold. If the new conformation is accepted, it is connected to its parent conformation and inserted into the grid. This process continues until a desired conformation is reached or a time limit is reached.

The sampling of grid cells is actually slightly more complicated than described above. For each grid cell the algorithm also keeps track of the number of neighboring grid cells that are empty (i.e., ones that contain no conformations). Non-empty grid cells with at least one empty neighbor grid cell are called *exterior* cells while the other non-empty grid cells are called *interior* cells. The sampling of grid cells is heavily biased towards exterior cells to improve the expansiveness of the conformational search. Note that the sampling bias towards low-density and exterior cells does not preclude exploration of higher-density and interior cells, albeit with a lower probability.

The overall behavior of the algorithm can be summarized as follows. The conformational sampling algorithm requires as input one or more known structures and a schema that defines the subsets of residues and associated moves. It then performs an expansive conformational search by iteratively applying a random move to a previously generated conformation. Conformations are selected inversely proportional to the local conformation density. This process can be considered an *undirected search*: the algorithm attempts to expand the tree of conformations equally in all directions. It is also possible to provide a goal conformation and have the search bias sampling with a small probability towards this goal conformation. This is called a *directed search*. (In robot motion planning, this is in fact the more common use case.) These modes of operation, directed and undirected search, can also be combined: a directed search can be performed first to find a transition between two conformations, and a transition envelope can be subsequently (or simultaneously) explored using an undirected search. Such generality allows for rapid exploration of conformational variability, both between and near known structures, as well as into unknown regions where experimentally unobserved (yet energetically stable) conformations may be hidden.

To enable analysis of extremely large systems, sims has been written to take advantage of all available computational resources (clusters, desktops, laptops) simultaneously and without special configuration. This is achieved by having each computational core perform a small run of sims and write the generated conformations back to a central database. The density estimates are then updated and a core can pick a random starting conformation from the database in a sparsely sampled part of the conformational space. Storing all generated data in a database also permits real-time analysis during a run.

## Results

In our computational experiments we explore how well sims performs with different schema s. The automatically-generated schema is defined as follows. There is a subset for each secondary structure element and one set containing all residues. Each loop, sheet, and helix has a sampling weight of 1.0, 0.2, and 0.1, respectively. With 9% probability the set with all residues is selected, while the remaining probability mass is distributed over the secondary structure elements proportional to their weight. The set of moves and their relative probabilities for each subset are the same: dihedral angle sampling, loop sampling, and rigid body movements are all sampled with equal probability. Note that loop sampling and rigid body movements are also applied to sheets and helices to allow these secondary structure elements to dissolve. However, since the sampling weight of loops is much larger, most of the conformational sampling is focused on loop changes. The energy (as a function of *all* backbone angles) is minimized 1% of the time. The expert-informed schema s described below either limit the degrees of freedom by only allowing moves for a small number of subsets or define additional subsets for residues whose motion need to be coordinated. In the first case, we can potentially explore the conformational space faster, but we risk eliminating a motion that is necessary for some conformational transition. In the second case, we simply encourage sampling particular degrees of freedom but do not sacrifice completeness of the algorithm.

In this work, all experiments were run on a multi-core cluster, typically using 200 cores. Though the times described in this section are measured in hours (assuming 200 cores) it should not be assumed that the problems necessarily represent 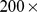 (number hours) cpu-hours of work. For small proteins, using many cores will lead to many parts of conformational space being visited independently by several cores, since the density estimates are updated infrequently when conformations are written in batches to a database. As we apply sims to larger protein complexes, this redundancy will become less of an issue as the probability of two cores exploring the same part of conformational space goes to 0 as the size of the conformational space increases. For the proteins below, it is still feasible to run sims on a standard desktop (and use less cpu time). For example, running an experiment from the Cyanovirin-N section on 16 cores (instead of 200) required a wall-time of 140 minutes (instead of 29 minutes), yielding essentially 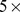 the compute time. Rigorously benchmarking and tuning the parallel performance would be a computationally intensive study and is beyond the scope of this work. In general, the actual wall time required in the experiments was slightly shorter than the estimated times reported here.

### Cyanovirin-N

It has been previously reported [27] that in Cyanovirin-N (cvn), primary flexibility arises from a *central hinge* spanning residues 45–55 and two secondary flex regions required for “breathing” flexibility that help overcome steric constraints in transitions between conformations. Based only on this preliminary expert knowledge we performed three separate experiments to further investigate the conformational flexibility of cvn, using schema s where backbone angles are allowed to change in (1) only the hinge, (2) the hinge and flex regions, or (3) all residues, respectively. In all three experiments the goal is to find a low-energy conformational transition between the monomeric (pdb:2EZM ) and domain-swapped (pdb:1L5E ) forms of cvn. We are interested in how fast sims can find paths with the different schema s, qualitative differences between the paths found and in identifying biophysically plausible paths in a neighborhood of the paths identified by sims.

**Figure 2:**
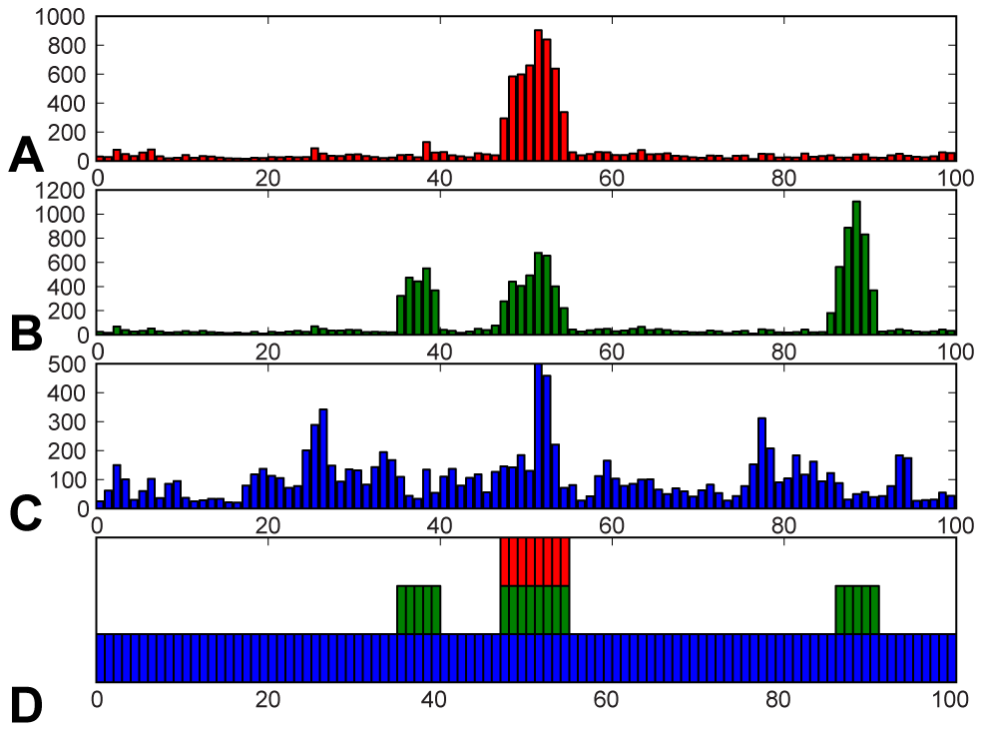
Plots of total angular change for each residue over the determined transition. (A) Central hinge only. (B) Hinge+flex region. (C) Automatic. (D) shows active residues explicitly used in planning for hinge (red), hinge+flex (green) and automatic (blue) runs.

**Figure 3:**
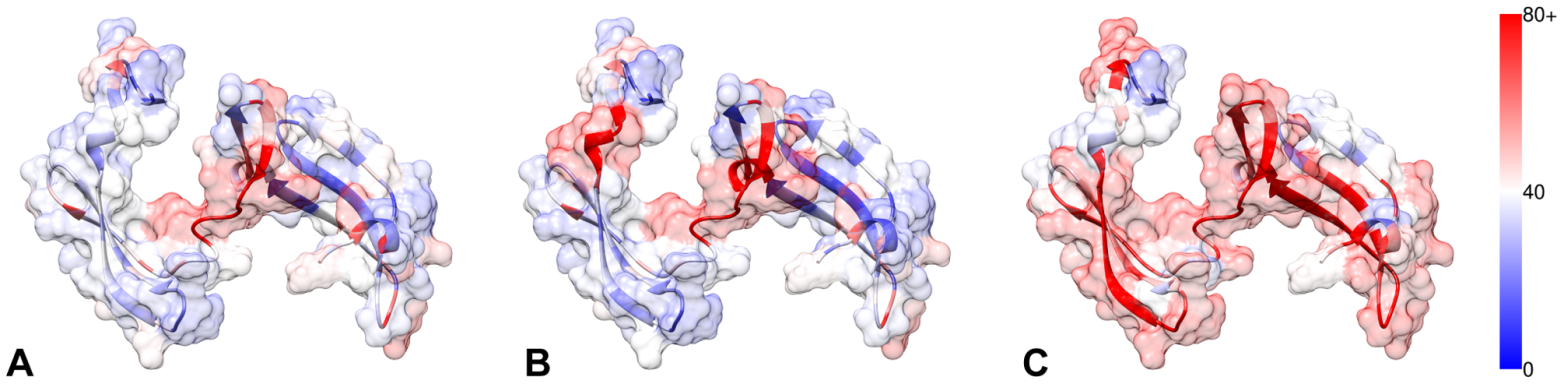
Plots of angular change in dofs for hinge (A), hinge+flex (B) and auto (C) experiments. Residues are colored by absolute total angular change, with blue indicating a small change and red a large change. Hinge and Flex (A, B) experiments show relatively low activity outside of the planning regions. The automatically guided experiment (C) shows high activity in the hinge and 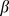-sheet regions of both subdomains.

The first experiment performed exploration exclusively in the central hinge region, with active dofs restricted to residue range 45–55 as in [27]. The schema used consisted of the default moves for residue range 45–55 and only a minimization move for the set of all residues. Though previous work [27] found this problem unsolvable when planning in the restricted residue range, a typical run in our setup was able to determine a transition between the monomeric and domain-swapped states in around 26 minutes. Analysis of the transition (see Figures 2A and 3A) shows essentially constant torsions outside of the range of 45–55, with insignificant changes in the ranges of 36–40 and 87–91 (previously [27] described as *flex* regions). However, as described later, one other region, 26–35, played a mildly significant role in this experiment, despite the fact that they were not explicitly used during the search as active dofs.

Though a feasible transition was determined using only the hinge region, subsequent analysis showed that restricting dofs strictly to the hinge region likely over-constrained the flexibility of the system, resulting in a long (qualitatively rough) transition between the start and goal states. It had also previously been shown [27] that, though the addition of dofs increases the size of the search space, planning with *flex* regions might ease the difficulty of this problem. The second cvn experiment therefore attempted planning on the expanded residue range, including both the central hinge and the previously described *flex* regions, residues 36–40 and 87–91. The schema used in this experiment comprised five subsets of residues, with sample probabilities in parentheses: the hinge region (0.16), each individual flex region (0.16 each), a subset containing the hinge region and both flex regions (0.50), and the set of all residues (0.01). Each subset has the default moves, except for the set of all residues, which only has a minimization move. This experiment took approximately 1.3 hours and showed almost identical torsional activity in the hinge region to the previous experiment, including 26–35 (see Figures 2 and 3). The flex regions, however, were very active in this run, though the expanded conformational freedom in these regions produced a 3-fold computational increase relative to the first experiment. The increase in computation time, combined with the findings from the first experiment – that the flex regions were not strictly required for solving this problem – appears to imply that the flex regions in fact do not play a significant role in the transition between monomeric and domain-swapped forms of cvn. This conclusion was reinforced by the results of the final experiment for cvn.

The final experiment for cvn involved a case where no expert knowledge was assumed. In this case, the automatic schema of dofs was applied to the system, alongside a second, overlapping subset of dofs composed of all residues – moves for this subset were sampled at 10% the rate of the automatically partitioned subset. This resulted in searching the full 198 dofs for cvn. Again a transition was determined, this time in around 30 minutes, a similar time to the first experiment, despite searching with a number of dofs nearly an order of magnitude greater than before. That is, though the hinge region performed a search using 20 dihedral angles and this experiment used 198, computation times were nearly identical. In this case, the transition determined employed nearly all torsional dofs, with the exception of those found within rigid sub-regions and, surprisingly, the flex regions (see Figures 2C and 3C).

All three runs were qualitatively similar at their start and end points, with the beginning of the paths defined by slow progression away from the highly-constrained starting state, and the end of the path characterized by alignment with the final position and a slow counter-rotation of the first and second half of the central hinge. The middle of the paths were relatively unconstrained with rotation mostly about the hinge. Qualitative transition smoothness was clearly the best for the auto- schema experiment, likely due to the availability of full conformational freedom.

Analysis of residue level torsional changes (Figure 2) for the three experiments revealed a number of common and unique features. Here, cumulative, residue-wise torsional change for residue 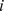 was calculated according to 
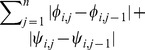
, where 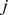 is the index of the conformation along the path. Unsurprisingly, the hinge region was active for all runs, though significantly less motion occurred in this region in the automatic run. As shown in the experiments, 26–35 represented secondarily important residues in all runs. More generally, the most active regions outside of the hinge for the auto- schema experiment were residue ranges 26–35 and 75–87, representing anti-complementary halves of sub-domains A and B respectively (i.e., one half of the 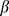-sheets defining these domains). It is clear from this analysis that the hinge region of CVN plays a dominant role in driving conformational transitions though, based on the results (and combined with the relative smoothness of the final experiment), there is also a large-scale sub-domain flexing that appears to aid this process.

**Figure 4:**
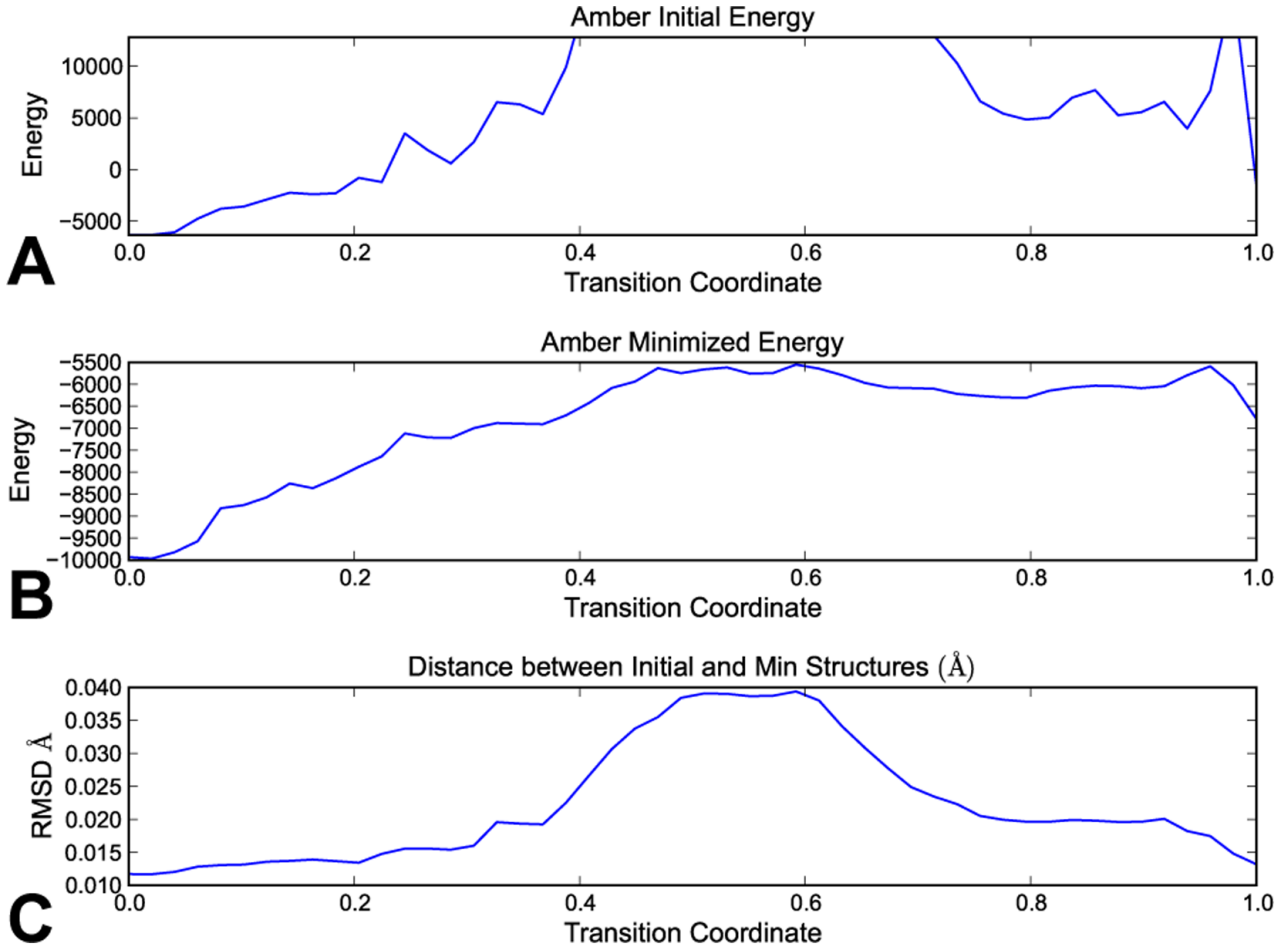
Energies as calculated by the Amber99 forcefield for a typical automatically guided run. All experiments produced similar plots. Energies are plotted against the transition coordinate (the amount of progress between start and goal for the transition). (A) Amber energies for the raw output of the automatically guided run (B) Amber energies after 100 rounds of minimization (C) Distance between raw output and minimized structure. All structures are determined to be of low energy, post-minimization, according to the Amber forcefield, with only mild (much less than 0.1Å full-atom rmsd) differences between the two structures.

Finally, all conformational transitions were analyzed using the Amber99 force field to calculate energies for the entire transition (Figure 4). Energies calculated for the raw output of sims were occasionally quite high, likely indicating some level of steric overlap between neighboring atoms. Using 100 steps of energy minimization always yielded an extremely low-energy structure, however. Further, the difference between the input and minimized structures were always less than 0.1Å full atom rmsd, essentially identical conformations. In fact, Figure 4 represents a typical plot for all subsequent experiments in this paper (i.e., including results for rbp and mbp ), with no minimized transition deviating significantly from the sims output.

In summary, sims was used in experiments above to investigate possible low-energy transitions between the monomeric and domain-swapped-versions of cvn. It was able to automatically determine active dofs and showed that, though expert knowledge can be used to rapidly determine solutions (as in the hinge experiment), incomplete knowledge (as in the flex experiment) can deleteriously bias results.

### Ribose-binding protein

rbp is known to exist in bound (pdb:1URP ) and unbound (pdb:2DRI ) states, reflected by the relative distance of two domains and the volume of the ligand binding space between them. Movement between the domains occurs via coordinated changes in three non-sequential loop regions connecting the two domains. The transition between the two forms of rbp seems relatively simple, given that the two conformations are only 4Å apart, but computationally producing energetically feasible transitions presents a formidable challenge (described more fully in the section *Motivating Problems*). Similar to the previous example, the goal is to compare an expert-determined schema with the default one. The expert-determined schema consist of two subsets of residues: one composed of the three loop regions and one with all residues. The former has the default moves associated with it (dihedral angle sampling, loop sampling, and rigid body movements, all sampled with equal probability) while the full set of all residues (sampled 1% of the time) only has an energy minimization move associated with it. This schema makes it possible to directly compare against results in a previous investigation [27]. While in [27] artificial distance constraints were required to prevent dissolution of the structure, we will demonstrate that sims can find a feasible transition with both the expert and the automatically-generated schemas.

The application of a modern planning algorithm for conformational exploration in this experiment led to extremely fast runtimes (on the order of seconds), producing energetically feasible transitions for all energy thresholds used by sims with both schema s. The final energetic threshold in the experiment presented was very close to the native energies of the start and goal states, yielding highly stable structures along the entire resulting conformational transition.

**Figure 5:**
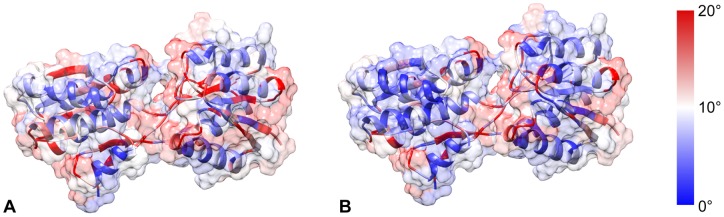
Plot of active dofs for expert (A) and auto (B) experiments. Color bar indicates cumulative per-residue torsional change over the entire determined transition. The two experiments show comparable activity in torsional dofs, largely confined to central loops through which much of the bending occurs.

Both domains remained coherent through the run, with only slight relative movements occurring in many of the 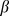-sheets and near the end of several helices in each domain observed relative to one another. Somewhat surprisingly, the transition determined by the expert guided run was essentially identical to the automatically guided run, with slightly more domain level variation occurring in the expert run (see Figure 5). Given the large differences in the number of dofs used and tightness of the energy constraint, it is very likely that both transitions represent slight variations of the minimum energy transition between these two states.

This experiment demonstrated that in spite of the significant kinematic challenge of making coordinated changes to non-sequential hinge residues using torsional dofs, sims is able to rapidly determine solutions using only unbiased energetic constraints, requiring no a priori knowledge.

### Maltose-binding protein

In [53] it was shown that a “hidden” energetically semi-stable conformation of mbp likely exists as an intermediate between known open and closed forms that has been only indirectly observed experimentally. Described as “semi-closed”, this distinct state is characterized by changes in the so-called *balancing interface*, a loop region that acts as a “spring” between the C-terminal and N-terminal domains. The goal of the experiments described here was to see if this hidden state could be determined using sims, when searching for a direct transition between open and bound forms of mbp. Changes between known open and bound forms of mbp represent a dominant bending deformation across the entire protein that involves changes in nearly all residues. As a result, no expert-determined set of active dofs was available for this system and an automatic schema was used. However, we will demonstrate that an initial run of sims can be used to determine active dofs. This is in itself may provide useful insight into the mechanism of mbp's function, but we will show that this can also be used to create a new schema that enables for a more rapid exploration of conformational space.

**Figure 6:**
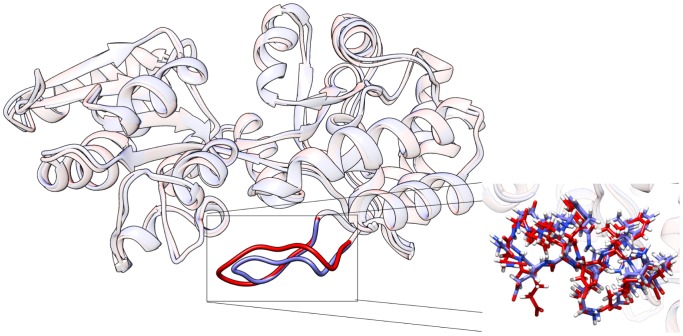
Comparison of the final state of the reverse transition (blue) and the direct transition (red). Structures are nearly identical save for a 10 residue relaxation of a loop region in the balancing interface. Both transitions come to within 1Å of their goal.

The first experiment used the default schema to find a transition from the unbound form (pdb:1OMP [73]) to the bound form (pdb:3MBP [84]). The search took approximately 15 hours to complete, coming to a state less than 1Å away from the goal – after which progress became significantly slower. The differences between the final state and the goal state were observed to occur almost exclusively around the end of the balancing interface loop region (Figure 6).

**Figure 7:**
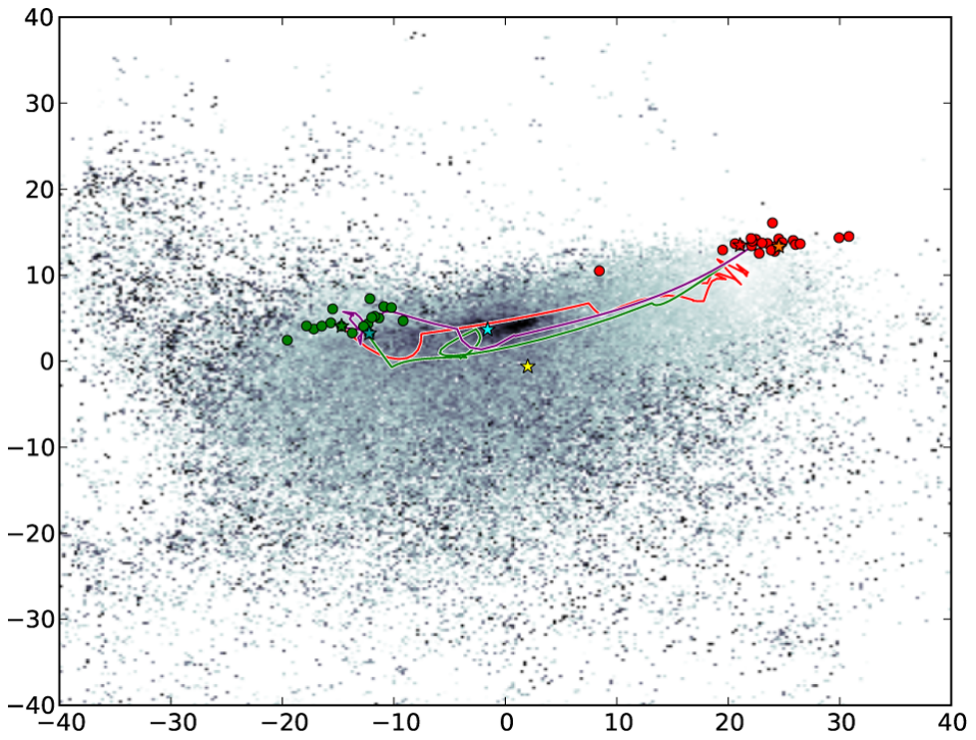
pca landscape of all conformations generated in the mbp experiments. Each point represents a unique conformation. The color indicates energy with darker colors representing more energetically stable states. The red path shows the path found with the default schema, starting from the open state with the bound state as the goal. The green path represents the reverse case, with the default schema, starting at the bound state and moving toward the open state. The purple path was produced using the expert- schema, moving from the bound state towards the open state. The yellow star indicates the position of a known nmr structure (pdb:2H25) of the semi-closed state. The aqua star indicates the centroid conformation of the energetic valley between the open and bound states and falls extremely close to all paths, as well as the nmr structure. The circular pattern in the green path was automatically generated and seems to arise from a slight bending reversal that occurs near the semi-closed state.

**Figure 8:**
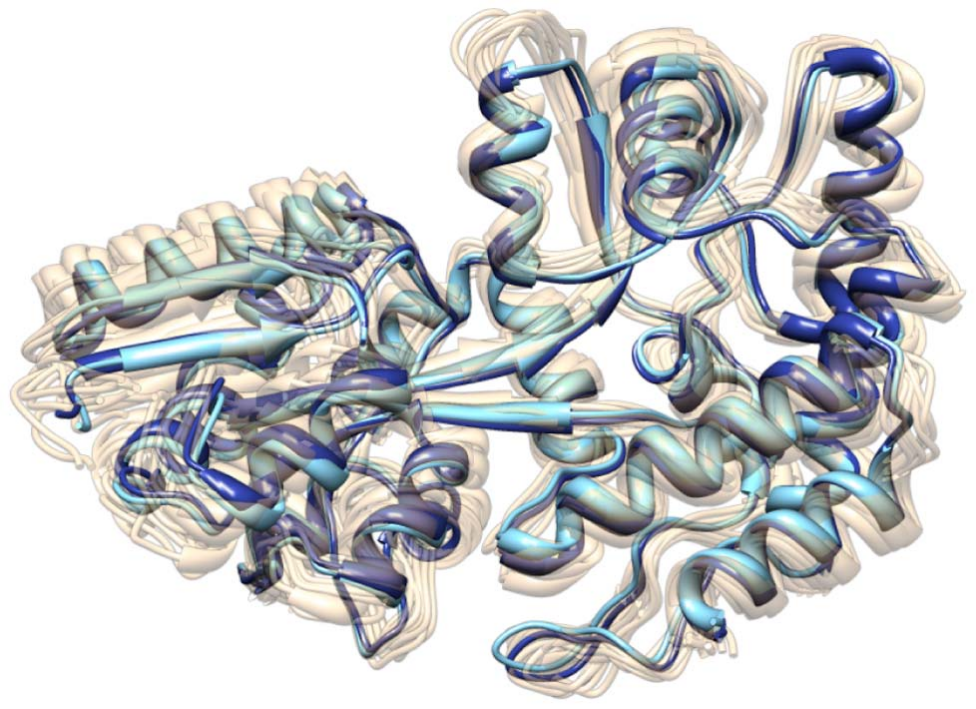
Comparison of a computationally identified intermediate state and nmr structure pdb:2H25. The identified intermediate state (dark blue) corresponds to the centroid of the low energy region shown in Figure 7. The nmr structure is known to be close to the semi-closed conformation of mbp, showing excellent agreement at the resolution of the ensemble. The closest state along a direct transition between known open and bound forms (aqua structure) of mbp to pdb:2H25 shows almost perfect agreement with the centroid structure.

To visualize how sims has explored the conformational space, we computed a low-dimensional embedding of all conformations using Principal Component Analysis (pca) [98]. Specifically, pca was applied to the Cartesian coordinates of all conformations generated during the search. By plotting each conformation as a point with coordinates given by the first two principal components we obtain a low-dimensional embedding of the conformations (see Figure 7). Similar to the results of [53], the open and bound forms of MBP were observed to cluster into two relatively tight groups, with the conformational transition (the red path in Figure 7) tracing a nearly direct transition between the two groups. Almost identical to the md results of [53], a large, relatively stable basin of intermediate conformations was observed almost directly between the bound and unbound groups. Calculating the centroid state of a representative set of low energy conformations from this basin yielded a structure that matched a known nmr structure [80] (pdb:2H25 ) for the “semi-closed” state of mbp to within the resolution of the experiment (Figure 8). Moreover, the low-energy conformational transition determined in this experiment was also found to pass extremely close to this state (to within less than 1Å full atom rmsd ), lending likelihood to the proposition that the semi-closed state of MBP represents a necessary transition intermediate between open and bound forms. These results were quickly determined using only an automatically generated schema, producing both a low-energy conformational transition between known states of mbp as well as a model for the semi-closed transition intermediate.

As is clear from energetic analysis of the input conformations (and basic biological intuition), the bound forms of mbp represent relatively high-energy conformations if the ligand is removed. The first piece of expert knowledge for the final experiments therefore involved reversing the start and goal states, starting instead at the bound form of mbp with a goal of reaching the unbound state – essentially removing the ligand and observing the energetic consequences. Using the same schema as before, the reversed search took approximately 5 hours to get within 1Å of the goal state (whereas the first took 15 hours). As in the previous case, the primary difference between the final state and the goal lay in the tip of the balancing interface (Figure 6), possibly due to energetic stabilizing factors in this region in the two forms, or an insufficiently resolved loop sampling schema in this region. Further, as before, the transition also passed within 1Å of the semi-closed state and traced an essentially direct transition between the bound form of MBP and the open group (green path in Figure 7).

For the final experiment we determined the active residues as measured by total torsional change per residue along the path found in the first experiment. The active residue ranges identified were: 101–104, 234–236, and 261–262. The schema we used, based on this information, included two subsets of residues: one with all the active residues (with the default set of moves) and the set of all residues (with only energy minimization, selected 1% of the time). With this schema it took approximately 2.5 hours to find a path from the bound to a state within 1Å of the unbound form. This path showed identical features to the previous two transitions (see purple path in Figure 7).

This collection of experiments demonstrated that, even absent expert knowledge about a protein system, sims can rapidly generate detailed information about low-energy conformational transitions. The conformational information generated during the search was also shown to be useful for conformational analysis, producing results typically requiring experiment or long-running md simulation. Finally, expert knowledge was generated from the initial investigation and subsequently used to generate information for new experiments. Besides dramatically improving experimental run times, this expert knowledge serves as a result in itself that could be applied as a constraint in future computational investigations by alternative methods.

## Discussion

In this work we have introduced a hybrid method for rapidly analyzing the conformational variability of proteins that combines all-atom energy calculations with abstractly defined long-range moves for conformational sampling. sims allows for rapid conformational exploration of input protein systems, producing an increasingly accurate sampling of the energetic landscape. While this method is not a replacement for md or approximate methods such as Normal Mode analysis, sims represents a powerful intermediate tool that benefits from aspects of both. Moreover, output from sims can easily be used as a launching point for more rigorous investigation using physics-based methods, reducing the substantial computational cost such investigation of long-range conformational variability would typically require.

We applied sims to three common classes of problems in computational biology: a hinge system, a non-sequential long-range correlated motion problem, and the discovery of a “hidden” conformational state of a protein. The demonstrated solutions to these problems were found rapidly and with minimal information as the result of a number of key features of the presented framework. The inclusion of a powerful schema based on collections of subsets of dofs, to aid successful move selection, simultaneously allowed the incorporation of expert knowledge while allowing likely active dofs to be rapidly explored.

We have shown that while this framework can benefit from expert knowledge when available, it is also capable of investigating systems about which little is known. In such cases automatic generation of a schema, as described earlier, can be used to perform initial explorations and, subsequently, determine active dofs from initial results. In the case of cvn, we showed a key example of how incomplete expert knowledge could negatively influence results and how automatic partitioning was used to refine this information.

Finally, we showed through the mbp experiments that, while not a replacement for md, sims can provide insight into a number of problems that have been traditionally studied by such methods. The ability to rapidly discover transient conformational intermediates (or at least to characterize a range of nearby neighbors), with minimal user input, presents a powerful extension to the range of analytical tools available to researchers.

### Future directions

Relative to the available computational power provided by the Rice University clusters (and eventually larger national computing clusters), the systems investigated here are likely far smaller than the limit of computationally tractability for this framework. Future studies will likely focus on significantly larger systems, or more complex problems (such as docking and protein-protein interaction).

While Rosetta proved both powerful and efficient for energy calculation and move generation, the move protocols used here were not necessarily tailored to the protein systems presented here. As the community continues to generate increasingly powerful move types, we hope to continuously extend the exploratory power of sims by including such developments into the framework.

Finally, the analysis performed on the datasets presented in this work, while extensive, only hints at the full range of options that could be used. Analyzing the graph-structure of the conformational exploration and casting the network as a Markov Process has previously demonstrated useful theoretical results [25], [31], [99]–[101]. Though pca was used for analysis of the conformational states generated, non-linear analysis of the energetic landscape [102] is another obvious direction for investigation. Most importantly for usability, however, will be a range of visualization output options, possibly benefiting from real-time (i.e., during data generation) interaction with intermediate results. It is expected that this improvement will likely provide the most important development for the computational biology community at large.

## Conclusions

The work has demonstrated the power and flexibility of a hybrid method for the investigation of protein conformational variability. Naturally integrating expert knowledge with automatic exploration allows both ease of use and the ability to account for partially known or uncertain information. This was demonstrated on a range of problem types for an array of commonly studied protein systems, showing sims' ability to rapidly provide answers for difficult problems related to conformational variability. Finally, sims represents both a tool for analysis and a launching point for further investigations by other methods, both theoretical and experimental.
